# Lower Cardiac Vagal Activity Predicts Self-Reported Difficulties With Emotion Regulation in Adolescents With ADHD

**DOI:** 10.3389/fpsyt.2020.00244

**Published:** 2020-04-17

**Authors:** Elisabet Kvadsheim, Ole Bernt Fasmer, Berge Osnes, Julian Koenig, Steinunn Adolfsdottir, Heike Eichele, Kerstin Jessica Plessen, Lin Sørensen

**Affiliations:** ^1^ Department of Biological and Medical Psychology, University of Bergen, Bergen, Norway; ^2^ Department of Clinical Medicine, University of Bergen, Bergen, Norway; ^3^ Haukeland University Hospital, Bjørgvin District Psychiatric Centre, Bergen, Norway; ^4^ Section for Experimental Child and Adolescent Psychiatry, Department of Child and Adolescent Psychiatry, Centre for Psychosocial Medicine, University of Heidelberg, Heidelberg, Germany; ^5^ University Hospital of Child and Adolescent Psychiatry and Psychotherapy, University of Bern, Bern, Switzerland; ^6^ Department of Visual Impairments, Statped West - National Service for Special Needs Education, Bergen, Norway; ^7^ Child and Adolescent Mental Health Center, Capital Region Psychiatry, Copenhagen, Denmark; ^8^ Department of Clinical Medicine, Faculty of Health Sciences, University of Copenhagen, Copenhagen, Denmark

**Keywords:** attention-deficit/hyperactivity disorder, heart rate variability, cardiac vagal activity, emotion regulation, autonomic nervous system, difficulties in emotion regulation scale

## Abstract

**Objective:**

To investigate the relation between cardiac vagal activity (CVA), a measure of autonomic nervous system (ANS) flexibility, and self-reported emotion regulation (ER) difficulties in adolescents with attention-deficit/hyperactivity disorder (ADHD) and controls.

**Methods:**

The sample comprised 11–17-year-old adolescents with ADHD (*n*=34) and controls (*n* = 33). Multiple linear regression analyses investigated the relation between CVA, as indexed by high frequency heart rate variability (HF-HRV), and ER difficulties as assessed by the Difficulties in Emotion Regulation Scale (DERS). Supplemental analyses were performed in ADHD and control groups separately. Analyses assessed effects of body mass index (BMI), physical activity levels, and HF peak as a surrogate of respiration on CVA.

**Results:**

Lower CVA was associated with ER difficulties, and specifically with limited access to effective ER strategies. When investigating the relation between CVA and ER in the ADHD and control groups separately, there was a tendency of lower CVA predicting limited access to effective ER strategies in the ADHD group, and not in the control group.

**Conclusion:**

The results suggest that lower CVA, i.e., reduced ANS flexibility, in adolescents with ADHD and controls is associated with self-reported ER difficulties, and specifically with limited access to effective ER strategies. There was a tendency for lower CVA to predict limited ER strategies only in the adolescents with ADHD and not controls.

## Introduction

Individuals with attention-deficit/hyperactivity disorder (ADHD) are characterized by difficulties with self-regulation (1), including emotion regulation [ER; (2)]. ER is the ability to influence which emotions one displays, at what time, and how they are experienced and expressed (3). In children and adolescents with ADHD, it is estimated that 25–45% have difficulties with ER (4). In healthy individuals, such ER difficulties are linked to inflexibility of the autonomic nervous system (ANS), often indexed by lower resting state cardiac vagal activity [CVA; (5–7)]. In ADHD, the link between ER difficulties and lower CVA is not as clear. This might be due to the reliance on parent-reports of ER difficulties in previous studies (8, 9). In the current study, we aimed to investigate the relation between self-reported ER difficulties and level of CVA in adolescents with ADHD.

Emotions—multifaceted processes involving changes in experiential, behavioral, and physiological responses (10)—are not always appropriate and in accordance with environmental demands, emphasizing the importance of ER for adaptive behavior and achievement of long-term goals (3). ER difficulties might be due to “bottom-up” psychological mechanisms such as orienting to emotional, salient stimuli and reward processing, or due to poorer “top-down” regulation (2). This might manifest as atypical allocation of attention to emotionally valenced stimuli, emotional lability (11, 12), and socially unsuitable behavior (2). The experience of emotions are associated with varying degrees of physiological arousal generated by the ANS (13). Flexibility of the ANS increases the capacity to rapidly modulate physiological and emotional states in accordance with situational demands. This flexibility is thought to depend on vagal modulation and to be reflected by CVA, derived from the variance in consecutive heart beat intervals (heart rate variability). CVA has been suggested as a transdiagnostic marker of ER difficulties (14). In healthy adults, self-reported ER difficulties have been associated with lower CVA, and specifically with difficulties with emotional impulse control, emotional clarity (15), and acceptance of negative emotions (16). Further, in a group of adolescents with and without psychiatric disorders (not ADHD), self-reports of difficulties with emotional awareness associated with lower CVA (17). Also of relevance, tendencies of worrying (18), anxiety disorders (19), and clinical depression in children and adolescents (20) are associated with lower CVA.

In ADHD, ER difficulties are shown to have a negative influence on well-being and self-esteem, peer relationships, school performance (21), and social skills (22). Further, such difficulties are linked to general anxiety disorder (23) and major depressive disorder [MDD; (24)], which are comorbid in about 20–30% of children and adolescents with ADHD [(22, 25)]. Only few studies have investigated the association between ER difficulties and CVA in children and adolescents with ADHD, and these have used parent-reports of ER difficulties. One such study found no association between CVA and ER difficulties (8), however another study found a dichotomous index of ER difficulties to be associated with lower CVA (9). Further, Griffiths et al. (26) found correlations of CVA with parent-reported symptoms of anxiety, social difficulties, and oppositional behavior in children and adolescents with ADHD. They concluded that CVA seem to reflect level of ER and adaptive behavior. However, no study has investigated whether CVA is associated with self-reported ER difficulties in children or adolescents with ADHD. As self-reports might capture internal, non-observable aspects of ER, they could provide information that parent reports do not detect. This is supported by a study in children and adolescents with ADHD, where self-reported symptoms of anxiety associated better with observed neurocognitive dysfunctions than parent- and teacher reports (27). Further, children with ADHD and controls have been found to report higher health-related quality of life (HRQoL) than HRQoL assessed by parent reports. This highlights the importance of also investigating self-reports in the study of psychophysiological aspects in children and adolescents with ADHD.

In the current study, we investigated the association between CVA and self-reported ER difficulties in adolescents with ADHD, and without ADHD (i.e. control group). We applied a naturalistic study design by including control participants with mental health disorders that often are comorbid to ADHD, such as anxiety disorders and Tourette Syndrome (TS). We hypothesized that lower CVA would be associated with ER difficulties, and expected this effect to be more prominent in adolescents with ADHD. We further explored the association between CVA and specific aspects of ER: acceptance of emotional responses, goal-directed behavior, and impulse control in relation to emotions, emotional awareness, access to effective ER strategies, and emotional clarity. We hypothesized that ER strategies would be the domain most closely associated with CVA, as CVA has been found to associate with neural activity during reappraisal and response modulation (28). This suggests that individuals displaying low CVA have difficulties with recruiting prefrontal brain areas necessary for modulation of amygdala activity during explicit ER. Further supporting our hypothesis, lower CVA has been associated with less use of constructive coping strategies (29) and interpersonal ER strategies such as support seeking (30).

## Methods

### Design

The current study is a cross-section of a follow-up project on ER in children with ADHD [studies from first wave: (31–33). The retention rate from the first assessment was 66.2% (ADHD= 60.7%, controls=71.7%); **Supplemental Figure 1**). The study protocol was approved by the Regional Committee for Medical Research Ethics of Western Norway (Study number: 2014/1304).

### Participants

The sample comprised 34 adolescents with ADHD and 33 controls between 11 and 17 years of age (**Table 1**). The majority of participants were male (*n*= 43; 64.2%). Children with suspected ADHD were originally referred from child and adolescent psychiatry units, and were assigned to either an ADHD or control group after identification with a semi-structured diagnostic interview: “the Schedule for Affective Disorders and Schizophrenia for School-Age Children – Present and Lifetime Version” [K-SADS; (34)]. Full-Scale IQ was measured with the Wechsler Intelligence Scale for Children—fourth version [WISC-IV; (35)]. In the follow-up assessment, the K-SADS was re-administered. All adolescents in the ADHD group still met the criteria for an ADHD diagnosis, except one participant having ADHD symptoms in remission (See **Table 2** for ADHD subtypes and comorbid disorders). Among participants in the control group, some comorbid conditions other than ADHD were present. Nineteen adolescents with ADHD were using stimulant medication. Parent reports on the DSM-IV ADHD rating scale [ADHD-RS; (36)] assessed the symptom distribution in the ADHD and control groups in both study assessments (37).

**Table 1:** Descriptive statistics for ADHD and control groups.

Variable	ADHD (*n* = 34)	Controls (*n* = 33)	t	χ2	*df*
Background information					
Gender (n, %)				.0080	1
Male	22 (64.7)	21 (63.6)			
Female	12 (35.3)	12 (36.4)			
Age (years)	14.34 (1.52)	14.62 (1.15)	.86		65
IQ	93.65 (10.27)	106.94 (10.63)	5.21**		65
BMI (kg/m^2^)	22.62 (5.73)	21.11 (2.91)	-1.37		49
Physical activity (times/week)	1.68 (.81)	2.70 (1.18)		16.68**	5
HF peak (Hz)	-1.41 (.25)	-1.36 (.21)	.85		65
Questionnaire scores					
DERS TOTAL	84.56 (23.83)	67.60 (15.36)	-3.47**		57
NONACCEPTANCE	11.05 (5.70)	8.19 (2.92)	-2.59*		50
GOALS	14.41 (5.57)	11.21 (4.42)	-2.60*		65
IMPULSE	13.82 (6.48)	9.33 (4.29)	-3.35**		57
AWARENESS	19.02 (4.83)	18.25 (5.10)	-.63		65
STRATEGIES	15.50 (6.75)	12.24 (4.09)	-2.40*		55
CLARITY	10.79 (3.71)	8.33 (2.79)	-3.06**		65
ADHD-RS	25.81 (10.86)	5.21 (6.67)	-9.38**		55
CVA (HF-HRV (n.u.))	1.78 (.12)	1.82 (.11)	1.55		65

ADHD, Attention Deficit Hyperactivity Disorder; BMI, Body Mass Index; HF peak, Peak of high frequency heart rate variability; DERS TOTAL, Difficulties in Emotion Regulation Scale total score; NONACCEPTANCE, Non-acceptance subscale; GOALS, Goals subscale; IMPULSE, Impulse control subscale; AWARENESS, Emotional awareness subscale; STRATEGIES, Emotion regulation strategies subscale; CLARITY, Emotional clarity subscale; ADHD-RS, ADHD Rating Scale; CVA, Cardiac Vagal Activity; IQ scores were collected in the first assessment of the study, when participants were 8-12 years old. *= p.<.05 **= p<.01.

**Table 2:** ADHD subtypes and comorbid diagnoses.

	ADHD (n, %)	Controls (n, %)
ADHD subtype		
IA	13 (38.2)	**-**
HI	1 (2.9)	**-**
C	20 (58.8)	**-**
TS	12 (35.3)	5 (15.2)
Anxiety disorders	10 (29.4)	2 (6.1)
ODD	7 (20.6)	–
OCD	2 (5.9)	1 (3.0)
MDD	1 (2.9)	–
Eneuresis	1 (2.9)	–
Epilepsy	1 (2.9)	–
Chronic motor tics	1 (2.,9)	–
Transient motor tics	1 (2.9)	–
Anorexia	–	1 (3.0)

IA, Inattentive subtype; HI, Hyperactive-impulsive subtype; C, Combined subtype; TS, Tourette Syndrome; ODD, Oppositional Defiant Disorder; OCD, Obsessive-Compulsive Disorder; MDD, Major Depressive Disorder.

### Measures

#### Cardiac Vagal Activity

CVA was derived from electrocardiogram (ECG) recordings. Participants were lying in a supine position and were instructed to relax while trying not to move or fall asleep. Participants were breathing spontaneously while the ECG was recorded for 6 minutes with a simple lead II setup at a sampling rate of 1,000 Hz. An A/D converter (Biopac, MP36, Biopac System Inc., Santa Barbara, CA) was used to obtain the signal, which was recorded with Biopac 4.0 BSL (Biopac Systems Inc. Santa Barbara, CA). The signal was conducted through three adhesive Ag/AgCl electrodes (T815 Dia. 55).

The first 5 minutes of the ECG recordings were subject to preparation and analysis in Kubios HRV version 2.2 (38). Inter-beat intervals (IBI) were calculated, and, if necessary, corrected. A total of 11 IBI corrections were made (.4–1.5% of IBIs in the corrected recordings), in six recordings (ADHD, *n* = 3; controls, *n* = 3). Additionally, one R-R interval containing an extra systole was removed from a recording.

Recordings were subject to a frequency analysis with the Fast Fourier Transformation, yielding a power spectrum displaying activity in the very low frequency (VLF, < .04 Hz), low frequency (LF,.04–.15 Hz) and high frequency (HF,.15–.4 Hz) domain (39). HF power was chosen as the applied measure of CVA (40). We assessed HF power in normalized units (n.u.), which corrects for the influence of VLF-HRV, and thus can be regarded as a measure of pure vagal influence (41). We used HF peak to indirectly control for respiratory frequency (42).

#### Emotion Dysregulation

The Difficulties in Emotion Regulation Scale [DERS; (43)] is a 36-item self-report questionnaire assessing ER difficulties. Items are rated on a five-point Likert-type scale from 1 (“almost never”) to 5 (“almost always”). In addition to a total score (DERS TOTAL), the questionnaire yields information on ER difficulties relating to different facets: (i) Non-acceptance of emotional responses (NONACCEPTANCE; e.g. “When I'm upset, I become embarrassed for feeling that way”; Cronbach's α in the current sample =.88); (ii) Difficulties engaging in goal-directed behavior (GOALS; e.g. “When I'm upset, I have difficulty focusing on other things”; α=.89); (iii) Impulse control difficulties (IMPULSE; e.g. “When I'm upset, I feel out of control”; α=.92); (iv) Lack of emotional awareness (AWARENESS; e.g. “I am attentive to how I'm feeling”; α=.75); (v) Limited access to ER strategies perceived as effective (STRATEGIES; e.g. “When I'm upset, it takes me a long time to feel better”; α=.85); and (vi) Lack of emotional clarity (CLARITY; e.g. “I am confused about how I feel”; α=.73). High scores reflect a high level of emotional dysregulation.

A Norwegian translation of the DERS was used (44) Although originally developed for adults, the DERS has been validated in adolescent samples, where it has shown satisfactory to excellent internal consistency (Cronbach's α=.72-.89) and adequate construct validity (45, 46). Further, it has been applied in a study of an adolescent ADHD sample (47).

In the current sample, six participants (ADHD: *n*=3, Controls: *n*=3) had one through five missing item scores, in total 14 missing scores.

#### Physical Activity and Body Mass Index (BMI)

Engagement in physical activity was assessed during the K-SADS, as the adolescents reported in “Other activities; Sport and Exercise”. Physical activity levels were scored on a six-point Likert-type scale from 0 (“zero times a week”) to 5 (“seven times or more a week”), as for Question 1 of the Physical Activity Questionnaire for Adolescents [PAQ-A; (48)]. The PAQ-A scoring norm was applied due to its convergent validity (49). Physical activity levels in between two categories were lowered to the nearest category. Further, BMI was calculated from measured height and weight as weight/height^2^ (kg/m^2^). BMI could not be calculated for four participants (ADHD, *n*=2; Controls, *n*=2), as they declined to get weighed or due to technical errors.

### Procedure

Adolescents and their parents participated in assessments conducted over two days. Test administrators were blinded to group status. On the first day, participants received information about the project and the assessments they would undergo. Then, ECG recordings were conducted between 9 a.m. and 1 p.m., to control for the effect of circadian variation on CVA (50). To rule out short-term effects of centrally stimulating medication for ADHD on heart rate (51) and ER, 89.5% of the adolescents with ADHD on stimulants (*n=*17, of 19) conducted a washout period of at least 24 h. This equals minimum five half-lives of the stimulant medication (52). On the second day, adolescents and their parents were separately interviewed with the K-SADS and filled out questionnaires. Participants received a reimbursement of £80 ($105).

### Statistical Analysis

Statistical analyses were performed in the Statistical Package for the Social Sciences version 24.0 (SPSS; IBM Corp., Armonk, NY, 2016). CVA data were log-transformed to approximate a normal distribution (53). We performed preliminary analyses with independent samples t-tests of group differences in distribution of age, gender, ADHD-RS scores, DERS scores, CVA, HF peak, Full-Scale IQ, and on HRV power spectrum values. Bivariate correlations were conducted between age, BMI, physical activity, HF peak, CVA, and DERS scores. No outliers, defined as values ± 3*SD* from the mean, were detected for DERS TOTAL, ADHD-RS, or CVA. Missing ADHD-RS data from one participant with ADHD was imputed with the sample mean. Missing BMI values were replaced with the sample mean and missing DERS values by the sample means of the respective items.

To test the hypothesis that lower CVA would be related to higher self-reported ER difficulties, we conducted multiple linear regression analyses. The DERS TOTAL score and the DERS subscale scores were used as dependent variables, respectively. CVA and diagnostic status were included as predictors. Analyses were adjusted for effects of age, gender, and HF peak. The regression model were adjusted for BMI [see (54)] and physical activity [see (55)] by including these as predictors, when a significant correlation between these variables and CVA and/or DERS subscale scores were found in the preliminary analyses. To adjust for multiple testing, we performed Bonferroni correction (56), yielding a corrected alpha level of *p* =.007 (.05/7). In follow-up regression analyses, an interaction term of ADHD*CVA (CVA scores were centralized with z scores for the interaction term) was added as a predictor to test for interaction effects between ADHD and CVA on ER difficulties (57, 58). As supplementary analyses, we conducted the same multiple linear regression analyses separately for the ADHD and control groups. We also performed follow-up analyses excluding participants in the control group with psychiatric disorders, to control for effects of a mixed participants control group.

## Results

### Preliminary Analyses

There were no differences in age nor gender between the ADHD and control group (**Table 1**). Parents reported higher ADHD-RS scores in the ADHD group compared to the control group. There were no significant differences in ADHD-RS scores between samples from the first and second assessment of the study. Further, control participants reported significantly higher physical activity levels than adolescents with ADHD did. There were no significant differences in BMI or HF peak between the groups. Bivariate correlation analyses showed that lower CVA was associated with higher DERS TOTAL, STRATEGIES, and CLARITY scores (**Supplemental Table 1**). Higher CVA also correlated with higher physical activity. CVA did not correlate with age nor BMI. Further, independent samples t-test showed no group difference in CVA. The ADHD group reported higher total scores and subscale scores on the DERS, except for on AWARENESS. No gender effects were found on CVA (**Supplemental Table 2**). The only gender effect that appeared in relation to DERS scores was that girls reported higher CLARITY scores than boys (Mean values: girls = 11.04; boys = 8.77; *t* = 2.67, *p=*.009). There were no group differences in CVA power spectrum values (**Supplemental Table 3**).

### Predicting Emotion Dysregulation by CVA

**Figure 1:**
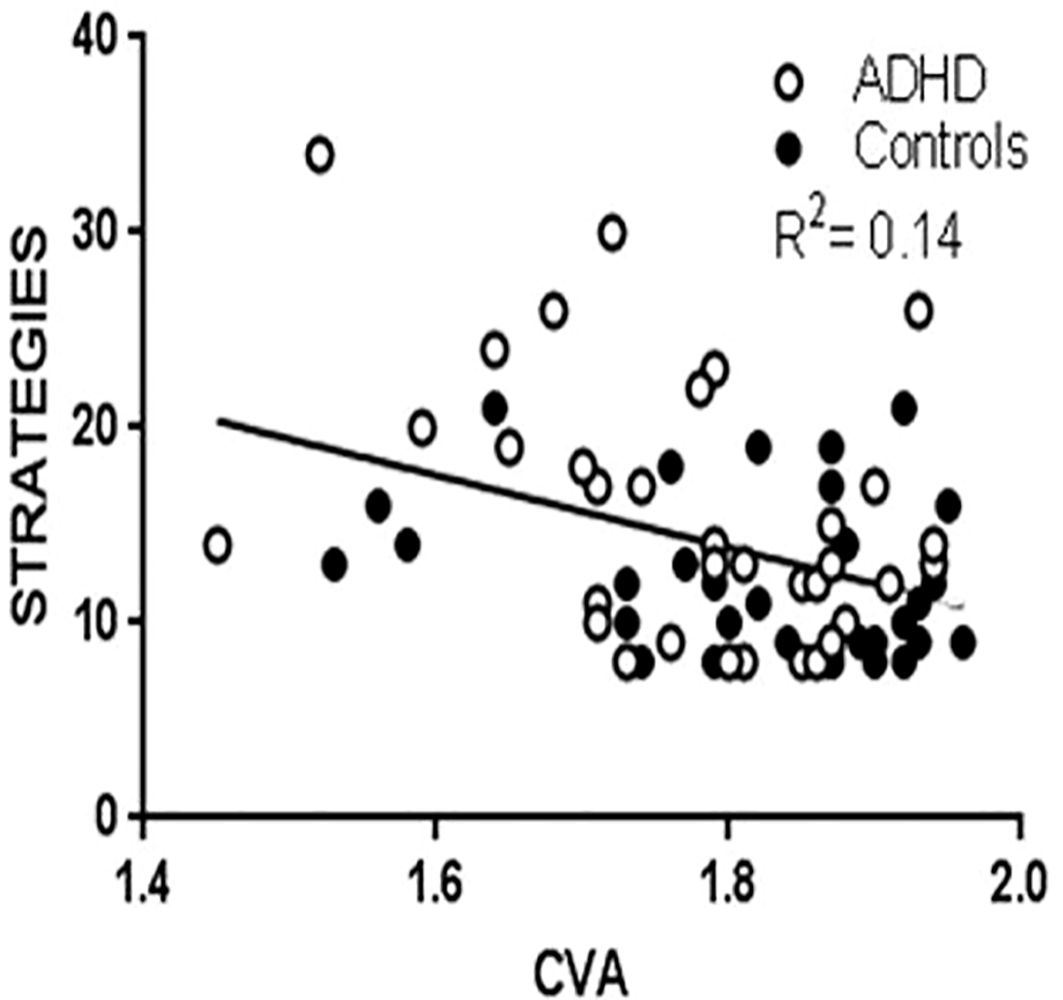
Scatterplot with regression line of STRATEGIES and CVA.

To test the effect of CVA on ER difficulties, we conducted multiple linear regression analyses (**Table 3**), which showed that lower CVA predicted higher STRATEGIES scores (**Figure 1**). There was a tendency for lower CVA to also predict higher DERS TOTAL scores. No effects of CVA were detected on NONACCEPTENCE, GOALS, IMPULSE, AWARENESS, or CLARITY. The diagnostic status of ADHD predicted higher scores on DERS TOTAL and all DERS subscales except for AWARENESS. Gender covaried with DERS TOTAL, STRATEGIES, and CLARITY, in that girls had higher scores. Higher HF peak covaried with higher CLARITY and GOALS. Neither age nor physical activity covaried with any of the DERS scores. Follow-up regression analyses adding the interaction term ADHD*CVA did not detect an interaction effect between ADHD and CVA on any of the DERS scores. Conducting the same multiple regression analyses separately in the diagnostic groups showed that lower CVA predicted higher STRATEGIES in the ADHD group (**Supplemental Table 4**; **Supplemental Figure 2**). CVA did not predict any other DERS scores in the ADHD group, and did not predict any DERS scores in the control group. Lastly, excluding controls with comorbid disorders, we found that CVA still predicted STRATEGIES (*p=*.004) in multiple linear regression analyses.

**Table 3:** Prediction of CVA on DERS scores.

DERS TOTAL					
Predictor	R^2^	df	F	p	β
ADHD	.311	(6, 66)	4.52*	.0020**	.39
Age				.89	.015
Gender				.021*	-.25
HF peak				.063	.21
Physical activity level				.69	.053
CVA				.019*	-.28
**NONACCEPTANCE**					
**Predictor**	**R^2^**	**df**	**F**	**p**	**β**
ADHD	.19	(6, 66)	2.33*	.017*	.32
Age				.67	.052
Gender				.057	-.23
HF peak				.17	.17
Physical activity level				.70	.055
CVA				.20	-.17
**GOALS**					
**Predictor**	**R^2^**	**df**	**F**	**p**	**β**
ADHD	.20	(6, 66)	2.41*	.030*	.29
Age				.92	.013
Gender				.12	-.18
HF peak				.043*	.25
Physical activity level				.81	-.033
CVA				.25	-.15
**IMPULSE**					
**Predictor**	**R^2^**	**df**	**F**	**p**	**β**
ADHD	.20	(6, 60)	2.43*	.0060**	.37
Age				.83	-.025
Gender				.19	-.15
HF peak				.50	.082
Physical activity level				.82	.032
CVA				.19	-.17
**AWARENESS**					
**Predictor**	**R^2^**	**df**	**F**	**p**	**β**
ADHD	.036	(6, 60)	.37	.39	.13
Age				.67	-.057
Gender				.78	.036
HF peak				.99	-.0010
Physical activity level				.27	.17
CVA				.34	-.12
**STRATEGIES**					
**Predictor**	**R^2^**	**df**	**F**	**p**	**β**
ADHD	.28	(6, 60)	3.82**	.064	.24
Age				.92	.011
Gender				.025*	-.25
HF peak				.13	.18
Physical activity level				.99	.000
CVA				.0030**	-.37
**CLARITY**					
**Predictor**	**R^2^**	**df**	**F**	**p**	**β**
ADHD	.35	(6, 60)	5.29**	.0030**	.37
Age				.30	.11
Gender				.0020**	-.33
HF peak				.015*	.27
Physical activity level				.772	.037
CVA				.050	-.23

ADHD, ADHD group diagnostic status; HF peak, Peak of high frequency heart rate variability; CVA, Cardiac Vagal Tone. *= p< .05 **= p< .01.

## Discussion

The aim of the present study was to investigate whether CVA, an index of autonomic flexibility, was associated with self-reported ER difficulties in adolescents with ADHD and controls. Results showed that, as expected, lower CVA significantly predicted higher STRATEGIES scores. There was also a tendency for lower CVA to predict higher DERS TOTAL scores. This indicates that level of CVA reflects the ability to use effective strategies for ER. Results also showed that the ADHD diagnosis associated with higher DERS scores, and thus higher ER difficulties, except for in relation to lack of emotional awareness. However, no interaction effects between level of CVA and ADHD on the DERS scores were found. When analyzing the association between CVA and DERS separately in the diagnostic groups, we found that lower CVA was associated with higher STRATEGIES scores in the ADHD group and not in the controls. Thus, despite the non-significant interaction effect between CVA and ADHD, there was a tendency for CVA to be associated with less access to effective ER strategies specifically in the ADHD group.

Our results suggest that level of CVA is important for the use of ER strategies perceived as effective by the individual experiencing and expressing the emotions. The STRATEGIES subscale measures the flexible use of situationally appropriate strategies (43). This finding is in line with a study of young adults, where higher CVA was associated with the use of constructive coping strategies during stressful events (29). Further, a study of adolescents found higher CVA to be associated with a tendency of seeking support from others in times of suffering, an important way to modulate and alleviate emotions (30). Lower CVA has been suggested to be associated with difficulties in recruiting prefrontal brain areas necessary for modulation of activity in amygdala during explicit ER, such as reappraisal and response modulation (28). Such prefrontal cortical regions have been found to display altered structural and functional maturation in ADHD (2, 4), and activity in these areas are hypothesized to be reflected in CVA (7). Through this network, inappropriate timing and magnitude of emotional experiences and behavioral reactions might be associated with reduced vagal modulation (5).

In our study, the small sample size, and as such low statistical power, may restrict the detection of interaction effects between ADHD and level of CVA on DERS scores. Despite this restriction, when looking into the relation between CVA and ER for the ADHD and control groups separately, we found a significant effect of CVA on ER (i.e., STRATEGIES) only in the adolescents with ADHD. This indicates that despite a lack of a significant interaction effect, there is a tendency of lower CVA to associate with higher ER difficulties specifically in the ADHD group. Since there are few studies of CVA and ER in ADHD, it is still not clear whether lower CVA predicts ER difficulties specifically in ADHD. As far as we know, only one previous study has found parent-reported emotion dysregulation to be associated with lower CVA in children and adolescents with ADHD (9). Further, another study found lower CVA in children and adolescents with ADHD to be associated with higher levels of anxiety, social difficulties, and oppositional behavior (26). It is possible that lower CVA could be related to ER difficulties in general, and not specifically for individuals with ADHD. In the current study, adolescents with ADHD reported higher levels of ER difficulties than controls, as also reported by parents in previous studies (2). It is therefore important to be aware of the association between ER difficulties and inflexible autonomic functioning among adolescents with ADHD, whether this association is specific for ADHD or not. This is because lower CVA might have implications for not only psychological, but also somatic health, morbidity, and longevity (59).

Previous studies of CVA and DERS in young, healthy adults have found CVA to be associated with less emotional impulse control and emotional clarity (15), and less acceptance of negative emotions (16). A longitudinal study over three years of children with and without psychiatric conditions found increasing CVA to be associated with fewer ER difficulties as measured by the DERS, and specifically with higher emotional awareness (17). The inconsistency in results between studies of CVA and DERS shows that it is not clear on a subscale level which specific ER domain are associated with CVA. In our study, we investigated a different age group than two of the previous studies. Our results are therefore not directly comparable to the previous findings.

Our results, indicating that lower CVA and thus inflexibility of the ANS is associated with limited access to ER strategies among adolescents with ADHD and controls, might have important theoretical and clinical implications. Our study contributes to the understanding of which specific domains of ER are related to CVA, which could support theoretical models of the relation between neural and psychological processes. Further, this might lead to increased focus on biofeedback treatment, which has been suggested to increase ER abilities [e.g. (60)]. Also, our finding of a significant association between level of CVA and self-reported ER difficulties shows that adolescents with ADHD seem to have insight into their own problems, which could be a good starting point for psychotherapeutic interventions. Further, future studies of adolescent ADHD samples might benefit from using self-reports instead of only observational reports from parents or teachers when investigating relations between ER difficulties and neurobiological variables such as CVA.

The current study has several strengths and limitations. One strength is that we have included possible confounders on CVA such as physical activity levels (55), BMI (54), and HF peak (42). Further, we controlled for circadian influences on CVA. Regarding limitations, we only used one questionnaire to assess ER difficulties, which may not capture the entire complexity of the concept. In addition, although we asked participants to conduct a washout period of ADHD medications to rule out short-term effects, we cannot rule out possible longer-term effects of such medication on CVA (51, 61, 62) and/or ER (63). Further, the mixed control group of adolescents with and without psychiatric diagnoses other than ADHD may have contributed to lower statistical power in detecting group differences. Finally, the study is cross-sectional in nature. Although there is evidence suggesting that lower CVA prospectively predicts emotion dysregulation (64), there is a possibility that difficulties in ER could influence CVA. Future research on the field would benefit from longitudinal designs, as well as studies designed to manipulate ER, to examine this notion.

## Data Availability Statement

Datasets are available on request. The raw data supporting the conclusions of this article will be made available by the authors, without undue reservation, to any qualified researcher.

## Ethics Statement

This study was carried out in accordance with the recommendations of Regional Committee for Medical Research Ethics of Western Norway, study number 2014/1304. All subjects gave written informed consent in accordance with the Declaration of Helsinki. The protocol was approved by the Regional Committee for Medical Research Ethics of Western Norway.

## Author Contributions

LS and KP designed the study. LS, SA, EK, and HE were involved in data collection. EK and LS, and BO performed data acquisition and analyses. EK and LS wrote the first draft, and JK, OF, BO, HE, and KP contributed to manuscript editing. All authors have read and approved the final manuscript.

## Funding

This work was supported by grants from the Research Council of Norway (190544/H110), the Western Norway Health Authority (MoodNet and the Network for Anxiety Disorders; 911435, 911607, 911827) to KP and the National Norwegian ADHD network to LS.

## Conflict of Interest

The authors declare that the research was conducted in the absence of any commercial or financial relationships that could be construed as a potential conflict of interest.

## References

[1] Barkley RA Behavioral inhibition, sustained attention, and executive functions: constructing a unifying theory of ADHD. psychol Bull (1997) 121:65–94. 10.1037/0033-2909.121.1.65 9000892

[2] Shaw P Stringaris A Nigg J Leibenluft E Emotion dysregulation in attention deficit hyperactivity disorder. Am J Psychiatry (2014) 171:276–93. 10.1176/appi.ajp.2013.13070966 PMC4282137 24480998

[3] Gross J Salovey P Rosenberg EL Fredrickson BL The Emerging Field of Emotion Regulation: An Integrative Review. Rev Gen Psychol (1998) 2:271–99. 10.1037/1089-2680.2.3.271

[4] Shaw M Watson B Sharp W Evans A Greenstein D Development of cortical surface area and gyrification in attention-deficit/hyperactivity disorder. Biol Psychiatry (2012) 72:191–7. 10.1016/j.biopsych.2012.01.031 PMC10376909 22418014

[5] Appelhans BM Leucken LJ Heart rate variability as an index of regulated emotional responding. Rev Gen Psychol (2006) 10:229–40. 10.1037/1089-2680.10.3.229

[6] Holzman JB Bridgett DJ Heart Rate Variability Indices as Bio-Markers of Top-Down Self-Regulatory Mechanisms: A Meta-Analytic Review. Neurosci Biobehav Rev (2017) 74:233–55. 10.1016/j.neubiorev.2016.12.032 28057463

[7] Thayer JF Lane RD A model of neurovisceral integration in emotion regulation and dysregulation. J Affect Disord (2000) 61:201–16. 10.1016/S0165-0327(00)00338-4 11163422

[8] Beauchaine TP Gatzke-Kopp L Neuhaus E Chipman J Reid MJ Webster-Stratton C Sympathetic- and parasympathetic-linked cardiac function and prediction of externalizing behavior, emotion regulation, and prosocial behavior among preschoolers treated for ADHD. J Consulting Clin Psychol (2013) 81:481–93. 10.1037/a0032302 PMC3952490 23544677

[9] Bunford N Evans SW Zoccola PM Owens JS Flory K Spiel CF Correspondence between Heart Rate Variability and Emotion Dysregulation in Children, Including Children with ADHD. J Abnormal Child Psychol (2017) 45, 1325–1337. 10.1007/s10802-016-0257-2 28032274

[10] Lang PJ The emotion probe: Studies of motivation and attention. Am Psychol (1995) 50:372. 10.1037/0003-066X.50.5.372 7762889

[11] Anastopoulos AD Smith TF Garrett ME Morrissey-Kane E Schatz NK Sommer JL Self-Regulation of Emotion, Functional Impairment, and Comorbidity Among ChildrenWith AD/HD. J Atten Disord (2011) 15:583–92. 10.1177/1087054710370567 PMC3355528 20686097

[12] Sobanski E Banaschewski T Asherson P Buitelaar J Chen W Franke B Emotional lability in children and adolescents with attention deficit/hyperactivity disorder (ADHD): clinical correlates and familial prevalence. J Child Psychol Psychiatry (2010) 51:915–23. 10.1111/j.1469-7610.2010.02217.x 20132417

[13] Levenson RW Blood, sweat, and fears: The autonomic architecture of emotion. Ann New Y. Acad Sci (2003) 1000:348–66. 10.1196/annals.1280.016 14766648

[14] Beauchaine TP Respiratory Sinus Arrhythmia: A Transdiagnostic Biomarker of Emotion Dysregulation and Psychopathology. Curr Opin Psychol (2015) 3:43–7. 10.1016/j.copsyc.2015.01.017 PMC4389219 25866835

[15] Williams DP Cash C Rankin C Bernardi A Koenig J Thayer JF Resting heart rate variability predicts self-reported difficulties in emotion regulation: a focus on different facets of emotion regulation: a focus on different facets of emotion regulation. Front Psychol. (2015) 6:261. 10.3389/fpsyg.2015.00261 25806017 PMC4354240

[16] Visted E Sørensen L Osnes B Svendsen JL Binder P-E Schanche E The Association between Self-Reported Difficulties in Emotion Regulation and Heart Rate Variability: The Salient Role of Not Accepting Negative Emotions. Front Psychol. (2017). 8:328 10.3389/fpsyg.2017.00328 28337160 PMC5343522

[17] Vasilev CA Crowell SE Beauchaine TP Mead HK Gatzke-Kopp LM Correspondence between physiological and self-report measures of emotion dysregulation: A longitudinal investigation of youth with and without psychopathology. J Child Psychol Psychiatry (2009) 50:1357–64. 10.1111/j.1469-7610.2009.02172.x 19811585

[18] Chalmers JA Heathers J.A.J Abbott M.J Kemp AH Quintana DS Worry is associated with robust reductions in heart rate variability: a transdiagnostic study of anxiety psychopathology. BMC Psychol (2016) 4: 32. 10.1186/s40359-016-0138-z 27255891 PMC4891851

[19] Sharma RK Balhara YP Sagar R Deepak KK Mehta M Heart rate variability study of childhood anxiety disorders. J Cardiovasc Dis Res (2011) 2:115–22. 10.4103/0975-3583.83040 PMC3144619 21814416

[20] Koenig J Kemp AH Beauchaine TP Thayer JF Kaess M Depression and resting state heart rate variability in children and adolescents—A systematic review and meta-analysis. Clin Psychol Rev (2016) 46:136–50. 10.1016/j.cpr.2016.04.013 27185312

[21] Riley AW Spiel G Coghill D Dopfner M Falissard B Lorenzo MJ , Factors related to health-related quality of life (HRQoL) among children with ADHD in Europe at entry into treatment. Eur Child Adolesc Psychiatry (2006) 15:I38–45. 10.1007/s00787-006-1006-9 17177014

[22] Bunford N Evans SW Becker SP Langberg JM Attention-deficit/hyperactivity disorder and social skills in youth: a moderated mediation model of emotion dysregulation and depression. J Abnormal Child Psychol (2015) 43:283–96. 10.1007/s10802-014-9909-2 PMC6538387 25037460

[23] Mennin DS Heimberg RG Turk CL Fresco DM Preliminary evidence for an emotion dysregulation model of generalized anxiety disorder. Behav Res Ther (2005) 43:1281–310. 10.1016/j.brat.2004.08.008 16086981

[24] Gross J Muñoz RF Emotion regulation and mental health. Clin psychol: Sci Pract (1995) 2:151–64. 10.1111/j.1468-2850.1995.tb00036.x

[25] Jarrett MA Ollendick TH A conceptual review of the comorbidity of attention-deficit/hyperactivity disorder and anxiety: implications for future research and practice. Clin Psychol Rev (2008) 28:1266–80. 10.1016/j.cpr.2008.05.004 18571820

[26] Griffiths KR Quintana DS Hermens DF Spooner C Tsang TW Clarke S Sustained attention and heart rate variability in children and adolescents with ADHD. Biol Psychol (2017) 124:11–20. 10.1016/j.biopsycho.2017.01.004 28099875

[27] Bloemsma JM Boer F Arnold R Banaschewski T Faraone SV Buitelaar JK Comorbid anxiety and neurocognitive dysfunctions in children with ADHD. Eur Child Adolesc Psychiatry (2013) 22:225–34. 10.1007/s00787-012-0339-9 23086381

[28] Steinfurth ECK Wendt J Geisler F Hamm AO Thayer JF Koenig J Resting State Vagally-Mediated Heart Rate Variability Is Associated With Neural Activity During Explicit Emotion Regulation. Front Neurosci (2018) 12:794. 10.3389/fnins.2018.00794 30455624 PMC6231057

[29] Fabes RA Eisenberg N Regulatory control and adults' stress-related responses to daily life events. J Pers Soc Psychol (1997) 73:1107–17. 10.1037/0022-3514.73.5.1107 9364764

[30] De Witte NA Sutterlin S Braet C Mueller SC Getting to the Heart of Emotion Regulation in Youth: The Role of Interoceptive Sensitivity, Heart Rate Variability, and Parental Psychopathology. PloS One (2016). 10.1371/journal.pone.0164615 PMC5065133 27741261

[31] Eichele H Eichele T Bjelland I Høvik MF Sørensen L Van Wageningen H Performance monitoring in medication-naïve children with Tourette Syndrome. Front Neurosci (2016) 10:50. 10.3389/fnins.2016.00050 26973443 PMC4771943

[32] Plessen KJ Allen EA Eichele H van Wageningen H Høvik MF Sørensen L Reduced error signalling in medication-naive children with ADHD: associations with behavioural variability and post-error adaptations. J Psychiatry Neurosci (2016) 41:77–87. 10.1503/jpn.140353 26441332 PMC4764484

[33] Sørensen L Sonuga-Barke E Eichele H van Wageningen H Wollschlaeger D Plessen KJ Suboptimal decision making by children with ADHD in the face of risk: Poor risk adjustment and delay aversion rather than general proneness to taking risks. Neuropsychology (2017) 31:119–28. 10.1037/neu0000297 27267090

[34] Kaufman J Birmaher B Brent D Rao U Flynn C Moreci P Schedule for Affective Disorders and Schizophrenia for School-Age Children-Present and Lifetime Version (K-SADS-PL): initial reliability and validity data. J Am Acad Child Adolesc Psychiatry (1997) 36:980–8. 10.1097/00004583-199707000-00021 9204677

[35] Wechsler D Wechsler intelligence scale for children-WISC-IV. San Antonio, TX: The Psychological Corporation. (2003).

[36] DuPaul G Power T Anastopoulos A Reid R ADHD Rating Scale-IV. Checklists, Norms and Clinical Interpretation. New York: Guilford (1998).

[37] Kvilhaug G Høygaard B Rønhovde T Aase H Eilertsen O Rydin S AD/HD Et verktøy for kartlegging av barn og ungdom. Oslo: Novus Forlag (1998).

[38] Tarvainen MP Niskanen JP Lipponen JA Ranta-Aho PO Karjalainen PA Kubios HRV–heart rate variability analysis software. Comput Methods programs biomed. (2014) 113:210–20. 10.1016/j.cmpb.2013.07.024 24054542

[39] Camm AJ Malik M Bigger J Breithardt G Cerutti S Cohen R for the Task Force of the European Society of Cardiology and the North American Society of Pacing and Electrophysiology. Heart Rate Variability: Standards of Measurement, Physiological Interpretation, and Clinical Use. Circulation (1996) 93:1043–65. 10.1111/j.1542-474X.1996.tb00275.x 8598068

[40] Koenig J Rash JA Kemp AH Buchhorn R Thayer JF Kaess M Resting State Vagal Tone in Attention Deficit (Hyperactivity) Disorder: A Meta-Analysis. World J Biol Psychiatry (2016) 18:1–33. 10.3109/15622975.2016.1174300 27073011

[41] Tarvainen MP Lipponen J Niskanen JP Ranta-aho PO Kubios HRV (ver. 3.3) User's guide. (2019).

[42] Thayer JF Sollers JJ Ruiz-Padial E Vila J Estimating respiratory frequency from autoregressive spectral analysis of heart period. IEEE Eng Med Biol Mag. (2002) 21:41–5. 10.1109/MEMB.2002.1032638 12222116

[43] Gratz K Roemer L Multidimensional Assessment of Emotion Regulation and Dysregulation: Development, Factor Structure, and Initial Validation of the Difficulties in Emotion Regulation Scale. J Psychopathol Behav Assess (2004) 26:41–54. 10.1023/B:JOBA.0000007455.08539.94

[44] Dundas I Vøllestad J Binder PE Sivertsen B The five factor mindfulness questionnaire in Norway. Scand. J Psychol (2013) 54:250–60. 10.1111/sjop.12044 23480438

[45] Neumann A van Lier PA Gratz KL Koot HM Multidimensional assessment of emotion regulation difficulties in adolescents using the Difficulties in Emotion Regulation Scale. Assessment (2010) 17:138–49. 10.1177/1073191109349579 19915198

[46] Weinberg A Klonsky ED Measurement of emotion dysregulation in adolescents. psychol Assess (2009) 21:616–21. 10.1037/a0016669 19947794

[47] Seymour KE Chronis-Tuscano A Halldorsdottir T Stupica B Owens K Sacks T Emotion regulation mediates the relationship between ADHD and depressive symptoms in youth. J Abnormal Child Psychol (2012) 40:595–606. 10.1007/s10802-011-9593-4 22113705

[48] Kowalski KC Crocker PR Donen RM The physical activity questionnaire for older children (PAQ-C) and adolescents (PAQ-A) manual. Saskatchewan: College of Kinesiology, University of Saskatchewan (2004).

[49] Kuczmarski RJ Ogden CL Grummer-Strawn LM Flegal KM Guo SS Wei R CDC growth charts: United States. Adv. Data (2000) 314:1–27. 10.1097/00008486-200203000-00006 11183293

[50] Malpas SC Purdie GL Circadian variation of heart rate variability. Cardiovasc Res (1990) 24:210–3. 10.1093/cvr/24.3.210 2346954

[51] Buchhorn R Conzelmann A Willaschek C Stork D Taurines R Renner TJ Heart rate variability and methylphenidate in children with ADHD. Attention deficit hyperactivity Disord (2012) 4:85–91. 10.1007/s12402-012-0072-8 22328340

[52] Ito S Pharmacokinetics 101. Paediatrics Child Health (2011) 16:535–6. 10.1093/pch/16.9.535 PMC3223885 23115489

[53] Ellis RJ Sollers JJ III Edelstein EA Thayer JF Data transforms for spectral analyses of heart rate variability. BioMed Sci Instrum (2008) 44:392–7. 19141947

[54] KoenigJJarczokMWarthMEllisRBachCHillecke et alT Body mass index is related to autonomic nervous system activity as measured by heart rate variability—a replication using short term measurements. J nutr. Health Aging (2014) 18:300–2. 10.1007/s12603-014-0022-6 24626758

[55] Gutin B Howe CA Johnson MH Humphries MC Snieder H Barbeau P Heart rate variability in adolescents: relations to physical activity, fitness, and adiposity. Med Sci sports Exercise (2005) 37:1856–63. 10.1249/01.mss.0000175867.98628.27 16286853

[56] Bland JM Altman DG Multiple significance tests: the Bonferroni method. Bmj (1995) 310:170. 10.1136/bmj.310.6973.170 7833759 PMC2548561

[57] Mayers A Introduction to statistics and SPSS in psychology. Boston (MA), Pearson Higher Ed. (2013).

[58] Tabachnick BG Fidell LS Ullman JB Using multivariate statistics. Boston, MA: Pearson (2007).

[59] Thayer JF Lane RD The role of vagal function in the risk for cardiovascular disease and mortality. Biol Psychol (2007) 74:224–42. 10.1016/j.biopsycho.2005.11.013 17182165

[60] Peira N Pourtois G Fredrikson M Learned cardiac control with heart rate biofeedback transfers to emotional reactions. PloS One (2013) 23:8 (7). 10.1371/journal.pone.0070004 PMC3720933 23894574

[61] Kim HJ Yang J Lee MS Changes of Heart Rate Variability during Methylphenidate Treatment in Attention-Deficit Hyperactivity Disorder Children: A 12-Week Prospective Study. Yonsei Med J (2015) 56:1365–71. 10.3349/ymj.2015.56.5.1365 PMC4541668 26256981

[62] Negrao BL Bipath P van der Westhuizen D Viljoen M Autonomic correlates at rest and during evoked attention in children with attention-deficit/hyperactivity disorder and effects of methylphenidate. Neuropsychobiology (2011) 63:82–91. 10.1159/000317548 21178382

[63] Rösler M Retz W Fischer R Ose C Alm B Deckert J Twenty-four-week treatment with extended release methylphenidate improves emotional symptoms in adult ADHD. World J Biol Psychiatry (2010) 11:709–18. 10.3109/15622971003624197 20353312

[64] Jandackova VK Britton A Malik M Steptoe A Heart rate variability and depressive symptoms: a cross-lagged analysis over a 10-year period in the Whitehall II study. psychol Med (2016) 46:2121–31. 10.1017/S003329171600060X 27181276

