# Supplementary material for: Lower Cardiac Vagal Activity Predicts Self-Reported Difficulties With Emotion Regulation in Adolescents With ADHD
Source: Front Psychiatry. 2020 Apr 17;11:244. doi: 10.3389/fpsyt.2020.00244 (PMC7181562; doi:10.3389/fpsyt.2020.00244)

Supplemental figure 1 Retention rates from the first wave of the study for the ADHD and control groups

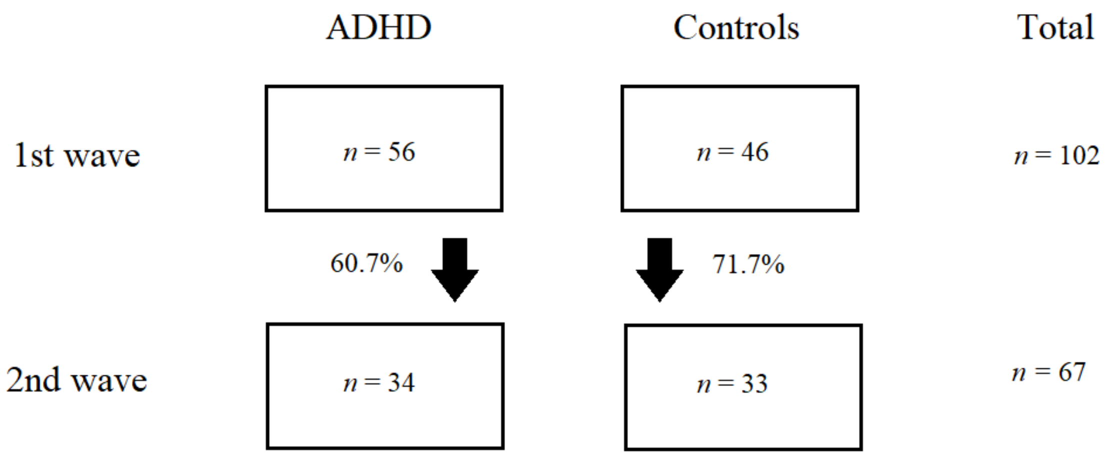

Supplement: Supplementary file 1 [file Image_1.pdf]
