# Supplementary material for: Lower Cardiac Vagal Activity Predicts Self-Reported Difficulties With Emotion Regulation in Adolescents With ADHD
Source: Front Psychiatry. 2020 Apr 17;11:244. doi: 10.3389/fpsyt.2020.00244 (PMC7181562; doi:10.3389/fpsyt.2020.00244)

Supplemental figure 2 Scatterplot with regression line of STRATEGIES and CVA for A) the ADHD group) and B) The control group.

A) **B)**

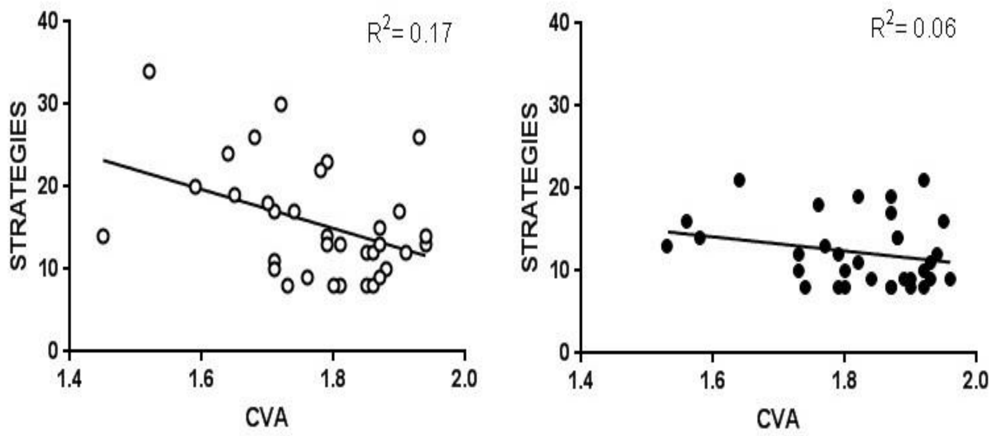

Supplement: Supplementary file 2 [file Image_2.pdf]
