# Supplementary material for: Lower Cardiac Vagal Activity Predicts Self-Reported Difficulties With Emotion Regulation in Adolescents With ADHD
Source: Front Psychiatry. 2020 Apr 17;11:244. doi: 10.3389/fpsyt.2020.00244 (PMC7181562; doi:10.3389/fpsyt.2020.00244)
Supplement: Supplementary file 3 [file Table_1.docx]

| **Supplemental Table 1** Bivariate correlations among main study variables | | | | | | | | |  |
| --- | --- | --- | --- | --- | --- | --- | --- | --- | --- |
| Total sample | | | | | | | | |  |
|  | CVA | 1 | 2 | 3 | 4 | 5 | 6 | 7 |  |
| Age | -.18 | .005 | .030 | -.011 | -.044 | -.049 | .034 | .088 |  |
| BMI | -.096 | -.16 | -.16 | -.14 | -.12 | -.073 | -.067 | -.10 |  |
| Physical activity level | .35** | -.19 | -.12 | -.17 | -.19 | .072 | -.21 | -.16 |  |
| HF peak | .22 | .12 | .11 | .17 | .012 | .0070 | .063 | .18 |  |
| ADHD-RS | -.16 | .25* | .16 | .21 | .21 | .18 | .13 | .22 |  |
| CVA |  | -2.9* | -.18 | -.16 | -.20 | -.08 | -.37** | -.24* |  |
| DERS |  |  |  |  |  |  |  |  |  |
| 1 TOTAL |  |  | .78** | .86** | .88** | .13 | .85** | .81** |  |
| 2 NONACCEPTANCE |  |  |  | .63** | .67** | -.17 | .66** | .62** |  |
| 3 GOALS |  |  |  |  | .75** | -.15 | .80** | .57** |  |
| 4 IMPULSE |  |  |  |  |  | -.002 | .72** | .64** |  |
| 5 AWARENESS |  |  |  |  |  |  | -.19 | .25* |  |
| 6 STRATEGIES |  |  |  |  |  |  |  | .56** |  |
| 7 CLARITY |  |  |  |  |  |  |  |  |  |
| ADHD group | | | | | | | | |  |
|  | CVA | 1 | 2 | 3 | 4 | 5 | 6 | 7 |  |
| Age | -.28 | .052 | -.015 | .035 | -.055 | .056 | .038 | .26 |  |
| BMI | -.027 | -.32 | -.22 | -.30 | -.32 | -.11 | -.15 | -.23 |  |
| Physical activity level | -.013 | .044 | .072 | -.13 | .13 | .24 | -.081 | .068 |  |
| HF peak | .077 | .28 | .24 | .29 | .22 | -.046 | .19 | .36* |  |
| ADHD-RS | .034 | -.22 | -.11 | -.13 | -.31 | .046 | -.16 | -.24 |  |
| CVA |  | -.27 | -.13 | -.11 | -.13 | -.078 | -.41* | -.29 |  |
| DERS |  |  |  |  |  |  |  |  |  |
| 1 TOTAL |  |  | .86** | .84** | .88** | -.019 | .87** | .75** |  |
| 2 NONACCEPTANCE |  |  |  | .72** | .73** | -.18 | .68** | .69** |  |
| 3 GOALS |  |  |  |  | .76** | -.36* | .78** | .48** |  |
| 4 IMPULSE |  |  |  |  |  | -.13 | .74** | .55** |  |
| 5 AWARENESS |  |  |  |  |  |  | -.23 | .15 |  |
| 6 STRATEGIES |  |  |  |  |  |  |  | .54** |  |
| 7 CLARITY |  |  |  |  |  |  |  |  |  |
| Control group | | | | | | | | |  |
|  | CVA | 1 | 2 | 3 | 4 | 5 | 6 | 7 |  |
| Age | -.10 | .049 | .29 | .0010 | .10 | -.17 | .14 | -.092 |  |
| BMI | -.16 | -.036 | -.23 | .014 | .15 | -.057 | -.020 | -.029 |  |
| Physical activity level | .53** | -.062 | -.040 | .053 | -.163 | .046 | -.12 | -.067 |  |
| HF peak | .367* | -.032 | -.059 | .10 | -.25 | .086 | -.096 | .026 |  |
| ADHD-RS | -.15 | .26 | .12 | .16 | .28 | .44* | -.055 | .27 |  |
| CVA |  | -.20 | -.12 | -.11 | -.18 | -.053 | -.237 | -.59 |  |
| DERS |  |  |  |  |  |  |  |  |  |
| 1 TOTAL |  |  | .46** | .85** | .81** | .29 | .75** | .83** |  |
| 2 NONACCEPTANCE |  |  |  | .30 | .33 | -.28 | .45** | .298 |  |
| 3 GOALS |  |  |  |  | .65** | .026 | .80** | .60** |  |
| 4 IMPULSE |  |  |  |  |  | .096 | .55** | .66** |  |
| 5 AWARENESS |  |  |  |  |  |  | -.24 | .35* |  |
| 6 STRATEGIES |  |  |  |  |  |  |  | .45** |  |
| 7 CLARITY |  |  |  |  |  |  |  |  |  |
|  |  |  |  |  |  |  |  |  |  |
| \|  \| \| --- \| |  |  |  |  |  |  |  |  |  |
|  |  |  |  |  |  |  |  |  |  |
|  |  |  |  |  |  |  |  |  |  |
|  |  |  |  |  |  |  |  |  |  |
|  |  |  |  |  |  |  |  |  |  |

Note. HF peak: Peak of high frequency heart rate variability; CVA: Cardiac Vagal Activity; DERS TOTAL: Difficulties in Emotion Regulation Scale total score; NONACCEPTANCE: Non-acceptance subscale; GOALS: Goals subscale; IMPULSE: Impulse control subscale; AWARENESS: Emotional awareness subscale; STRATEGIES: Emotion regulation strategies subscale; CLARITY: Emotional clarity subscale; ADHD-RS: ADHD Rating Scale; BMI: Body Mass Index. Table displays Pearson's Correlation Coefficient. *= *p*<*.*05; ** = *p*<.01.
