# Supplementary material for: Lower Cardiac Vagal Activity Predicts Self-Reported Difficulties With Emotion Regulation in Adolescents With ADHD
Source: Front Psychiatry. 2020 Apr 17;11:244. doi: 10.3389/fpsyt.2020.00244 (PMC7181562; doi:10.3389/fpsyt.2020.00244)
Supplement: Supplementary file 4 [file Table_2.docx]

Supplemental table 2 Gender differences in CVA and DERS

	Boys	Girls	*t*
CVA	1.80 (.11)	1.80 (.13)	.12
DERS TOTAL	72.39 (19.66)	83.04 (23.89)	.197
NONACCEPTANCE	8.90 (3.92)	10.97 (5.77)	1.57
GOALS	12.16 (4.95)	14.04 (5.67)	1.42
IMPULSE	10.98 (5.91)	12.75 (5.88)	1.18
AWARENESS	18.82 (4.95)	18.32 (5.02)	-.39
STRATEGIES	12.87 (5.21)	15.75 (6.42)	2.00
CLARITY	8.77 (2.95)	11.04 (3.96)	2.67**

Note. CVA: Cardiac Vagal Activity; DERS TOTAL: Difficulties in Emotion Regulation Scale total score; NONACCEPTANCE: Non-acceptance subscale; GOALS: Goals subscale; IMPULSE: Impulse control subscale; AWARENESS: Emotional awareness subscale; STRATEGIES: Emotion regulation strategies subscale; CLARITY: Emotional clarity subscale. Df = 65. *= p.<.05 **= p<.01
