# Supplementary material for: Lower Cardiac Vagal Activity Predicts Self-Reported Difficulties With Emotion Regulation in Adolescents With ADHD
Source: Front Psychiatry. 2020 Apr 17;11:244. doi: 10.3389/fpsyt.2020.00244 (PMC7181562; doi:10.3389/fpsyt.2020.00244)
Supplement: Supplementary file 5 [file Table_3.docx]

Supplemental table 3 Power spectrum analysis for ADHD and control groups

	ADHD	Controls	*t*
VLF	2.91 (.40)	2.87 (.30)	-.46
LF (ms2)	2.88 (.46)	2.81 (.42)	-.59
LF (n.u.)	1.54 (.20)	1.44 (.23)	-1.85
HF (ms2)	3.11 (.51)	3.19 (.44)	.64

Note. Table displays values from power spectrum analysis of heart rate variability. VLF: Very Low Frequency; LF (ms2): Low Frequency in ms2; LF (n.u.): Low Frequency in normalized units; HF (ms2): High Frequency in ms2 HF (n.u.): High Frequency in normalized units. Df = 65. *= p<.05 **= p<.01
