# Supplementary material for: Lower Cardiac Vagal Activity Predicts Self-Reported Difficulties With Emotion Regulation in Adolescents With ADHD
Source: Front Psychiatry. 2020 Apr 17;11:244. doi: 10.3389/fpsyt.2020.00244 (PMC7181562; doi:10.3389/fpsyt.2020.00244)
Supplement: Supplementary file 6 [file Table_4.docx]

Supplemental table 4 Prediction of CVA on DERS scores in diagnostic subgroups

**ADHD**					
DERS TOTAL					
Predictor	R^2	df	F	p	β
Age	.48	(5, 33)	1.68	.85	-.034
Gender				.15	-.25
HF peak				.097	.29
Physical activity level				.87	-.029
CVA				.16	-.26
NONACCEPTANCE					
Predictor	R^2	df	F	p	β
Age	.14	(5, 33)	.95	.79	-.28
Gender				.19	-1.34
HF peak				.20	1.33
Physical activity level				.99	.012
CVA				.51	-.68
GOALS					
Predictor	R^2	df	F	p	β
Age	.21	(5, 33)	1.51	.81	-.044
Gender				.10	-.29
HF peak				.087	.30
Physical activity level				.25	-.21
CVA				.61	-.094
IMPULSE					
Predictor	R^2	df	F	p	β
Age	.11	(5, 33)	.68	.64	-.092
Gender				.51	-.12
HF peak				.22	.23
Physical activity level				.72	.067
CVA				.44	-.15
AWARENESS					
Predictor	R^2	df	F	p	β
Age	.11	(5, 33)	.70	.63	.096
Gender				.33	.18
HF peak				.73	-.062
Physical activity level				.13	.29
CVA				.69	-.078
STRATEGIES					
Predictor	R^2	df	F	p	β
Age	.32	(5, 33)	2.65	.50	-.12
Gender				.10	-.28
HF peak				.15	.23
Physical activity level				.29	-.17
CVA				.020*	-.41
CLARITY					
Predictor	R^2	df	F	p	β
Age	.31	(5, 33)	2.53	.25	.20
Gender				.15	-.24
HF peak				.043*	.34
Physical activity level				.76	.050
CVA				.23	-.21

**Controls**					
		DERS TOTAL			
Predictor	R^2	df	F	p	β
Age	.13	(5, 32)	.81	,98	.0050
Gender				.11	-.34
HF peak				.69	.086
Physical activity level				.42	.20
CVA				.10	-.41
		NONACCEPTANCE			
Predictor	R^2	df	F	p	β
Age	.13	(5, 32)	.79	.15	.30
Gender				.35	-.20
HF peak				.58	.12
Physical activity level				.87	.040
CVA				.42	-.20
		GOALS			
Predictor	R^2	df	F	p	β
Age	.067	(5, 32)	.39	.99	.0010
Gender				.43	-.17
HF peak				.47	.16
Physical activity level				.46	.19
CVA				.23	-.31
		IMPULSE			
Predictor	R^2	df	F	p	β
Age	.090	(5, 32)	.53	.92	.020
Gender				.49	-.15
HF peak				.43	-.18
Physical activity level				.94	.018
CVA				.55	-.15
		AWARENESS			
Predictor	R^2	df	F	p	β
Age	.066	(5, 32)	.38	.34	-.20
Gender				.48	-.15
HF peak				.84	.045
Physical activity level				.43	.21
CVA				.36	-.23
		STRATEGIES			
Predictor	R^2	df	F	p	β
Age	.10	(5, 32)	.61	.60	.11
Gender				.33	-.21
HF peak				.79	.060
Physical activity level				.77	.076
CVA				.19	-.34
		CLARITY			
Predictor	R^2	df	F	p	β
Age	.25	(5, 32)	1.82	.46	-.14
Gender				.0070*	-.56
HF peak				.64	.094
Physical activity level				.34	.22
CVA				.13	-.35

Note. ADHD: ADHD group diagnostic status; HF peak: Peak of high frequency heart rate variability; CVA: Cardiac Vagal Activity. *= p< .05 **= p< .01
